# Achieving Digestive Autonomy and Gastrointestinal Continuity in a Patient with Short Bowel Syndrome Secondary to Concomitant Jejunal Atresia and Small Intestinal Hirschsprung's Disease

**DOI:** 10.1055/a-2351-9413

**Published:** 2024-07-16

**Authors:** Alejandro R. Velasquez, Thomas O. Xu, Yu-Ting Liu, Sulaiman Kidwai, Teresa L. Russell, Laura Tiusaba, Krystal Artis, Anthony Sandler, Andrea Badillo, Marc A. Levitt

**Affiliations:** 1 Department of Colorectal and Pelvic Reconstruction, Children's National, Washington, District of Columbia, United States; 2 The George Washington University School of Medicine and Health Sciences, Washington, District of Columbia, United States; 3 Department of Surgery, Colorectal and Pelvic Reconstructive Surgery, Children's National Hospital, Washington, District of Columbia, United States; 4 Department of Surgery, The George Washington University School of Medicine and Health Sciences, Washington, District of Columbia, United States

**Keywords:** STEP procedure, total colonic Hirschsprung's disease, short bowel syndrome, Hirschsprung's pull-through, jejunal atresia

## Abstract

Concomitant presentation of jejunal atresia and Hirschsprung's disease is rare and places children at high risk for developing short bowel syndrome and parenteral nutrition dependence, which can affect the feasibility/timing of pull-through. A patient was born with jejunal atresia with a delayed diagnosis of Hirschsprung's disease. After several procedures and bowel resections, the patient was ultimately left with an end jejunostomy and long Hartman's pouch with short bowel syndrome, dependent on parenteral nutrition. The patient initially presented to our institution at age 2 with failure to thrive secondary to an obstructed/dilated jejunostomy and mild enterocolitis of their defunctionalized segment. The patient subsequently underwent completion of subtotal colectomy and revision of jejunostomy utilizing a serial transverse enteroplasty to manage the dilated bowel and gain length. The patient was able to wean off parenteral nutrition and achieve nutritional autonomy by age 5. Following this, the patient was able to undergo an ileoanal pull-through. After the pull-through, the patient was able to pass stool independently and suffered no major complications to date. Serial transverse enteroplasty can be successfully utilized in patients with a history of Hirschsprung's disease and jejunal atresia to achieve nutritional autonomy and ultimately reestablish gastrointestinal continuity with pull-through.

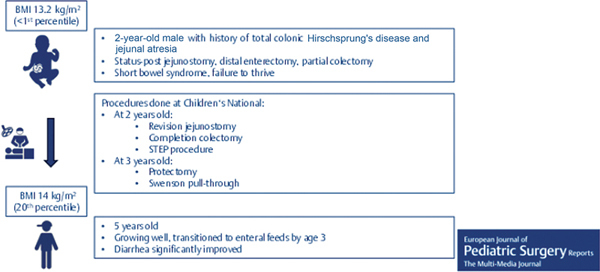

## Introduction

Hirschsprung's disease (HD) is a common cause of neonatal obstructive bowel disease characterized by the congenital absence of ganglion cells. The transition of aganglionic to ganglionated bowel most frequently occurs in the rectosigmoid colon; however, longer segment variants involving the entire colon, like total colonic HD and small intestinal Hirschsprung's disease (SIHD), occur less frequently and only represent 2 to 13% of all cases of HD. 1 2 Jejunal–ileal atresia is another common cause of neonatal bowel obstruction, thought to be caused by intrauterine vascular compromise. 3 Simultaneous occurrence of both is extremely rare 4 5 6 placing these children at high risk for short bowel syndrome (SBS) and its associated complications. 7 8 With this rare combination, the SBS can affect the possibility and the timing of a pull-through.

Herein, we present a case of jejunal atresia associated with a delayed diagnosis of SIHD resulting in SBS, which was successfully managed with resection of the distal aganglionic small and large bowel and a serial transverse enteroplasty (STEP) resulting in achieving digestive autonomy and reestablishment of gastrointestinal continuity with a successful jejunal–anal pull-through, with bowel control.

## Case Report

A 2-year-old term male was referred after initial management at another institution. At birth, he was reportedly diagnosed with sigmoid volvulus and jejunal atresia, whereby he underwent jejunostomy and mucous fistula creation and devolving of the sigmoid. At 2 months of age, the patient underwent a jejunostomy reversal at the outside institution; however, they developed a distention and feeding intolerance concerning a distal obstruction and requiring recreation of the jejunostomy. Prior to reversal, a rectal biopsy was performed and confirmed HD. At the time of jejunostomy reversal, the patient underwent colonic mapping, which confirmed total aganglionosis to the level of the distal jejunum. The jejunostomy was opened more proximally at the ganglionic level, and distal enterectomy and a near-total colectomy were performed, leaving him with 90 cm of small bowel proximal to the ileostomy and a 10-cm Hartmann's pouch. The reasoning for leaving Hartmann's pouch by the previous institution is unknown. Postoperatively, the patient developed SBS dependent on parenteral nutrition (PN). The patient otherwise had no other reported comorbidities.

**Fig. 1:**
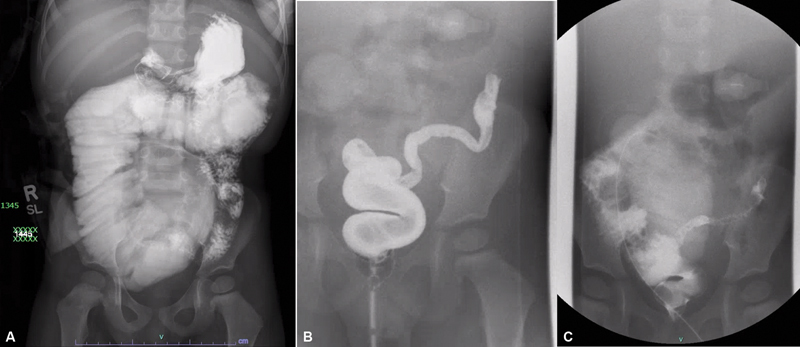
( **A** ) Fluoroscopy showing dilated jejunal loop proximal to the jejunostomy occupying the entire central abdomen. In addition, there is residual colon to the splenic flexure. ( **B** ) Contrast enema showing residual segment of narrowed descending colon and proximal sigmoid colon. ( **C** ) Contrast enema confirming dilated jejunal loops proximal to jejunostomy found on prior fluoroscopy.

At 2 years of age, the patient's family presented to our institution for continued intestinal rehabilitation. The patient initially presented with dehydration, electrolyte imbalance, and poor enteral (gastrostomy tube) intake. He weighed 14.6 kg. He was found to have a significantly dilated proximal jejunal loop on initial upper gastrointestinal fluoroscopy ( Fig. 1A ). Retrograde contrast enema showed a narrowed segment of retained descending colon and proximal sigmoid colon ( Fig. 1B ). A contrast study via the jejunostomy confirmed the previously identified dilated jejunal loops, which was measured to be about 7 cm on the initial fluoroscopy ( Fig. 1C ). The patient was then taken for endoscopic evaluation, which demonstrated a stricture of the jejunostomy site and moderate inflammation in the rectum and jejunum. Additionally, biopsies were taken from the jejunostomy site and confirmed the presence of ganglion cells. Based on these findings, we proceeded with revision jejunostomy to address the stricture and completion colectomy to address the enterocolitis noted in the defunctionalized segment. Given the patient's dilated distal jejunum that required resection, massively dilated bowel and PN-dependent short gut, a STEP procedure was performed to taper the bowel.

**Fig. 2:**
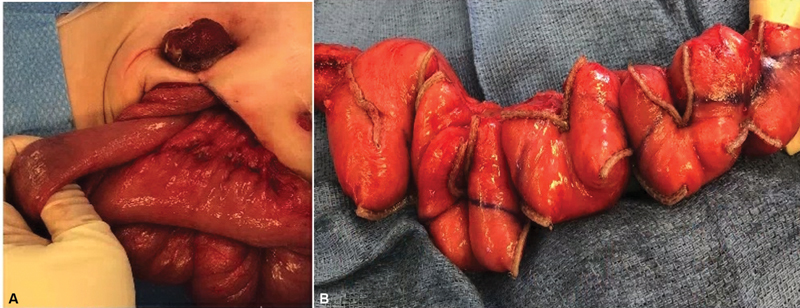
( **A** ) Jejunum twisted 360 degrees as it entered the jejunostomy. ( **B** ) Result of STEP procedure on dilated jejunum, allowing for narrowing and lengthening of dilated colon. STEP, serial transverse enteroplasty.

At the time of surgery, a 360-degree twist of the jejunum proximal to the ostomy was incidentally identified, which was likely causing poor intake and the dilation previously described ( Fig. 2A ). The entire small bowel length was measured at 124 cm with the dilated portion of proximal jejunum comprising 30 cm of the total length. The STEP of the dilated segment of jejunum enabled the simultaneous narrowing and lengthening of the dilated segment to 45 cm for a total small bowel length of 139 cm ( Fig. 2B ). A very short Hartman's pouch was left at the level of the peritoneal reflection.

The patient recovered well from the procedure. By the age of 3, 1 year out from his procedure, the patient had weaned off PN to full enteral formula feeds. At 5 years of age, the patient had a body mass index of 14 kg/m ^2^ (20 ^th^ percentile), weight of 22 kg (90 ^th^ percentile), and height of 123 cm (99.874 ^th^ percentile) and returned to the clinic to discuss feasibility of pull-through and reestablishment of gastrointestinal continuity. At this time, he had also weaned off enteral formula and was taking a regular diet by mouth with only gastrostomy tube use for fluid support and small intestinal bowel overgrowth antibiotic prophylaxis. Furthermore, the patient had developed a thickening of his jejunostomy output.

Given the patient's progress, we felt he had met criteria for a pull-through, i.e., good growth, off total PN and enteral supplemental feeds, and with thickened stoma output. We therefore proceeded with completion of a proctectomy and jejunal–anal pull-through. The initial dissection for proctectomy was completed in a Swenson-like fashion. The jejunostomy was taken down and the remainder of the rectal dissection was carried out intraabdominally. A total of 140 cm of bowel was measured, and after mobilization of the small bowel, the jejunum was pulled through.

The patient recovered uneventfully in the hospital and was discharged with a constipating diet (such as applesauce, white flour, and gradually adding fats after no stool accidents for 48 hours), fiber, and loperamide to manage his expected hypermotility. On 1-month follow-up, on an exam under anesthesia, his anastomosis was well healed. He had severe perianal rash 1 month postoperatively that was improved with further management of his hypermotility and application of polymeric cyanoacrylate solution and zinc oxide-based diaper cream. At 2 months postoperatively, the patient's stooling frequency and stool consistency have improved to 10 to 12 stools per day from 20+ stools per day immediately postoperatively. The perianal rash has reduced to small redness between the buttocks. He has not had any episodes of enterocolitis.

## Discussion

We present a case of jejunal atresia and SIHD resulting in SBS that required chronic PN. To our knowledge, this is the first reported case of STEP being used to ultimately allow a child to achieve nutritional autonomy and become a suitable candidate for pull-through. Ileal atresia and HD are relatively common causes of neonatal obstruction; however, concomitant occurrence of both is rare with few reported cases. 4 6 9 10 11 12 While the exact etiology is unknown, it is believed that the vascular insult within the 6 ^th^ to 8 ^th^ week of life that is presumed to cause ileal atresia could also interrupt the caudal migration of neural crest cells/ganglion cells resulting in both total colonic and small intestinal aganglionosis distal to the site of atresia. 12

It is critical to recognize these cases because an unrecognized distal obstruction threatens any anastomosis made during the treatment of jejunal atresia. Similarly, to previously reported cases, 5 6 12 13 our patient was only diagnosed with HD after problems occurred following the small bowel anastomosis. Failure to resect affected distal bowel can lead to anastomotic leakage or persistent obstruction necessitating additional interventions. In our patient, failure to recognize the HD diagnosis led to an unnecessary jejunostomy reversal and recreation in a patient already with a limited amount of small bowel.

Patients with SIHD essentially have the most severe form (type 1) of SBS, given the absence of the terminal ileum, ileocecal valve, and colon. 14 Payen et al demonstrated that patients with at least 80 cm of small bowel have the potential to achieve digestive autonomy, whereas nearly all those with less remained dependent on PN, some requiring intestinal transplantation. 7

This case was challenging as at time of referral to our center, we were presented with a patient with failure to thrive, intestinal failure, and reportedly 90 cm of remaining small bowel. Several other factors contributed to this patient's poor initial status. He had an obstructed jejunostomy with a massively dilated distal jejunum, which not only prevented any progress on enteral feeds, but also posed a risk for bacterial overgrowth. Additional revision on an already short length of remaining bowel risks even worse nutritional outcomes. Furthermore, the patient had their defunctionalized aganglionic bowel posing a potential inflammatory nidus, which may have been contributing to this patient's poor clinical progress. It has been well demonstrated that outcomes (such as rates of enterocolitis in the excluded segment) have been associated with a longer extent of aganglionosis as represented in our case. 7 15 16 17

Our solution was to utilize the STEP procedure, which produced excellent results. First introduced by Kim et al in 2003, 18 the STEP procedure has been shown to be effective at increasing bowel length, tapering dilated bowel, improving absorptive capacity, and facilitating weaning of PN. 19 20 In this case, it provided an excellent way to take advantage of the dilated jejunum that allowed for lengthening of the patient's bowel. Removing the residual aganglionosis likely also helped. The interventions spared this patient from long-term nutritional dependency. Not only was he able to wean from PN, but the patient was also able to demonstrate adequate growth and the ability to produce thickened stools, which allowed for definitive pull-through.

At the time of follow-up, the patient continues to do well without signs of enterocolitis, with decreasing stool frequency per day, no perianal skin excoriation, and bowel control. Those who have undergone a pull-through for total colonic aganglionosis often experience perianal dermatitis and excoriation due to the high frequency of bowel contents; however, this can be treated with antimotility agents (loperamide, with adjuncts including cholestyramine, hyoscyamine, and Lomotil [diphenoxylate hydrochloride and atropine sulfate]), a skincare protocol based on the level of skin breakdown (including cyanoacrylate-based barrier liquid, zinc-based diaper cream), and a constipating diet with good effect. 21 Our patient was initially started on 4 mg Imodium twice a day but complained of chest pain. The dose was reduced to 2 mg twice a day, which was better tolerated from a stool consistency and chest pain standpoint. At last visit, the patient was also started on ½ tsp psyllium fiber daily. Family was counseled on local wound management with consistent wound washing with mild soap and water as well as fresh dressing changes with which they were able to follow well.

## Conclusion

Concomitant jejunal atresia and small intestinal HD is a rare entity but places the child at high risk for SBS given the residual amount of functional small intestine. Achieving nutritional autonomy becomes more difficult the longer the extent of aganglionosis; however, we report the first case of the use of the STEP, which successfully led to a child achieving nutrition autonomy and ultimate restoration of gastrointestinal continuity.

## Notes

Importance for a Pediatric Surgeon Jejunal atresia associated with small intestinal Hirschsprung's disease is rare. It may result in short bowel syndrome requiring long-term parenteral nutrition, which can affect the timing of the Hirschsprung's pull-through. We describe the successful use of resection of the aganglionic small and large bowel as well as a serial transverse enteroplasty in such a patient who was then able to achieve digestive autonomy and ultimately reestablishment of gastrointestinal continuity with a successful jejunal–anal pull-through.
